# A potential mechanism for low tolerance feedback loops in social media flagging systems

**DOI:** 10.1371/journal.pone.0268270

**Published:** 2022-05-26

**Authors:** Camilla Jung Westermann, Michele Coscia

**Affiliations:** IT University of Copenhagen, Copenhagen, Denmark; University of Pisa, ITALY

## Abstract

Many people use social media as a primary information source, but their questionable reliability has pushed platforms to contain misinformation via crowdsourced flagging systems. Such systems, however, assume that users are impartial arbiters of truth. This assumption might be unwarranted, as users might be influenced by their own political biases and tolerance for opposing points of view, besides considering the truth value of a news item. In this paper we simulate a scenario in which users on one side of the polarity spectrum have different tolerance levels for the opinions of the other side. We create a model based on some assumptions about online news consumption, including echo chambers, selective exposure, and confirmation bias. A consequence of such a model is that news sources on the opposite side of the intolerant users attract more flags. We extend the base model in two ways: (i) by allowing news sources to find the path of least resistance that leads to a minimization of backlash, and (ii) by allowing users to change their tolerance level in response to a perceived lower tolerance from users on the other side of the spectrum. With these extensions, in the model we see that intolerance is attractive: news sources are nudged to move their polarity to the side of the intolerant users. Such a model does not support high-tolerance regimes: these regimes are out of equilibrium and will converge towards empirically-supported low-tolerance states under the assumption of partisan but rational users.

## Introduction

Social media has become one of the primary means of consumption of information online [1–3]. However, the barriers for creating content on social media are low and have given rise to a wave of misinformation [4–7]. As a reaction, social media platforms have pledged to contain the spread of fake news. One common strategy is to use crowdsourced flagging systems: users will flag news items and those receiving more than their fair share of flags will be fact-checked by experts.

This system works in theory, but the objectivity of users in evaluating the truthfulness of a news item has been called into question. Phenomena like confirmation bias [8, 9], echo chambers [10, 11]—although the magnitude of their effect has been called into question [12, 13] –, trust in the source or lack thereof [14], information overload [15] might pollute such objectivity—for a variety of different reasons that are not necessarily only ascribed to social identity. As a result, users might be prone to give a pass to misinformation confirming their world view, while at the same time being excessively zealous against news challenging their opinion. In a previous study, we developed an agent-based model where this potential differential treatment is taken into account: in such a model, the crowdsourced flagging systems produce counterproductive results [16, 17]. Most flags end up assigned to truthful and neutral news sources, while producers of polarizing misinformation were barely flagged.

In this paper, we bring our agent-based experiment one step further. We are interested in the effect of differential tolerance. In the original model, all users had the same level of tolerance—which determines how far from their worldview a news item must be to earn a flag from them. While it is true that both liberals and conservatives are prone to characterize opposing points of view as fake news [18], it is questionable whether they do so at the same level of ideological distance. In fact, there is evidence that some portions of the opinion spectrum might be less tolerant than others—e.g. one side having more and stronger reasons to tolerate opposing points of view [19]; media on one side of the spectrum using stronger outrage language than the other [20]; or users reacting more or less strongly when exposed to opposing points of view [21].

We start by assigning a lower tolerance to agents on one side of the polarization spectrum. In such a scenario, news sources on the opposite side of the intolerant users receive more flags. In an extension of the model where we allow sources to react to these flags by changing their polarity to minimize the amount of flags received, we see that sources are attracted to move towards the polarization values of the intolerant users. This result is less trivial that might appear at first sight: maximally intolerant users flag everything near their position, thus they are repulsive for everything that is not exactly conforming to their polarity. In fact, in our model the tolerance sweet spot is different from zero.

Moreover, tolerance is not an absolute and immutable quality of an individual, but can change in different contexts [22], for instance increasing when talking in abstract terms, but decreasing when facing concrete examples [23]. In other research, greater democratic activism is linked with an increase of political tolerance [24].

In a second extension of our model we hypothesize that users on social media might copy the low-tolerance strategy from users on the other side of the polarity spectrum, in a form of retaliation—which is a classic game-theoretic strategy. There is research supporting retaliation as a realistic potential mechanism: male group members are more likely to retaliate against an outgroup if the outgroup makes them question their own identity (such as in a political debate) [25]. Intergroup anger is a group-level emotion that predicts the desire of the individual to harm a threatening outgroup as a whole [26]. This is a group-level example of appraisal theory [27] which shows that a person with a strong perception of their own self would tend to retaliate against other individuals threatening that self [28]. In this case, it works at a group level when group affiliation is incorporated in the image of the self, as it is the case for many groups. This process of group integration in a self image is known and studied as “self-categorization theory” [29].

Besides literature-backing we can find examples of opposite ends of the political spectrum copying each other strategies as a form of retaliation. For instance, the derogatory term “snowflake” has been widely used by conservatives to mock the variety of issues triggering a strong emotional response from liberals [30], but has been quickly retorted against conservatives exploiting their own triggering issues (https://www.theguardian.com/commentisfree/2017/jan/16/snowflake-in-chief-donald-trump, date of access November 16th, 2021). Similar fate occurred to the “Make America Great Again” (MAGA) meme, accusing the outgroup to have caused the downfall of a country, a fall that can be reverted by strong actions from the ingroup. Originally a campaign slogan for Donald Trump [31], it received a response with the same underlying message (“We just did”) by the Biden campaign (https://www.gq.com/story/joe-biden-we-just-did-hat, date of access November 16th, 2021).

In reverse, the aggressive “say her name” stance taken to highlight police brutality against black women [32] had been co-opted by the right to attack the left about the killing of Ashli Babbit in the Jan 6th 2021 Capitol Riot (https://www.newsweek.com/say-her-name-used-memorialize-ashli-babbitt-draws-backlash-online-over-phrases-origin-1559867). In another example, brigades of alt-right social media users weaponized the methods of cancel culture [33]—through which many conservatives had been asked or forced to step down from their positions due to claims from liberals of racism/sexism/homophobia—to ostracize liberal celebrities. One example is the firing of movie director James Gunn (https://www.dailydot.com/parsec/james-gunn-firing-alt-right/ date of access November 16th, 2021).

In our models, this retaliation process creates downward tolerance spirals: a tolerant society in our model is out of equilibrium and will settle on a significantly lower average tolerance level as a result of users attempting to attract news sources on their side by exploiting the flagging system. Again, this tolerance is not zero, confirming that there are more complex dynamics at play than simply minimizing tolerance to maximize the number of flags assigned to opposing points of view. Interestingly, our model settles this equilibrium tolerance in an interval that is empirically supported: the equilbrium parameter range is included in the range that is the best at reproducing the relationship between a news source popularity and the number of flags it receives on Facebook, as described in previous work [16].

This study is fully based on simulations on an agent-based model, thus its conclusions should be verified with empirical experimentation in future works. However, ABMs have been successfully applied to social media polarization studies in the past and have proven their usefulness [9, 16, 34–39]. Our ABM is designed to capture the most salient characteristics of social media information consumption: echo chambers; selective exposure; confirmation bias; realistic distributions of user, source polarity, and the popularity of news sources; realistic topology for the social network among users with communities, high clustering, broad degree distributions, and small world effect. We support our choices by identifying the relevant pieces of literature in the sections describing the model.

The code necessary to reproduce our results is freely available at http://www.michelecoscia.com/?page_id=2018.

## Materials and methods

We start by describing the Bipolar model, which is the basic model from our previous work. Then, we describe our main variation of the Bipolar model at the basis of our results: the Relative model. Finally, we explain the two further extensions to the Relative model supporting our discussions: the Relative model with evolving source polarity, and with evolving user tolerance.

### Original model: Bipolar model

The basic model is the Bipolar model introduced in [16]. We describe it here briefly, and we refer to the original paper for a more in-depth explanation and motivation of the components.

**Fig 1:**
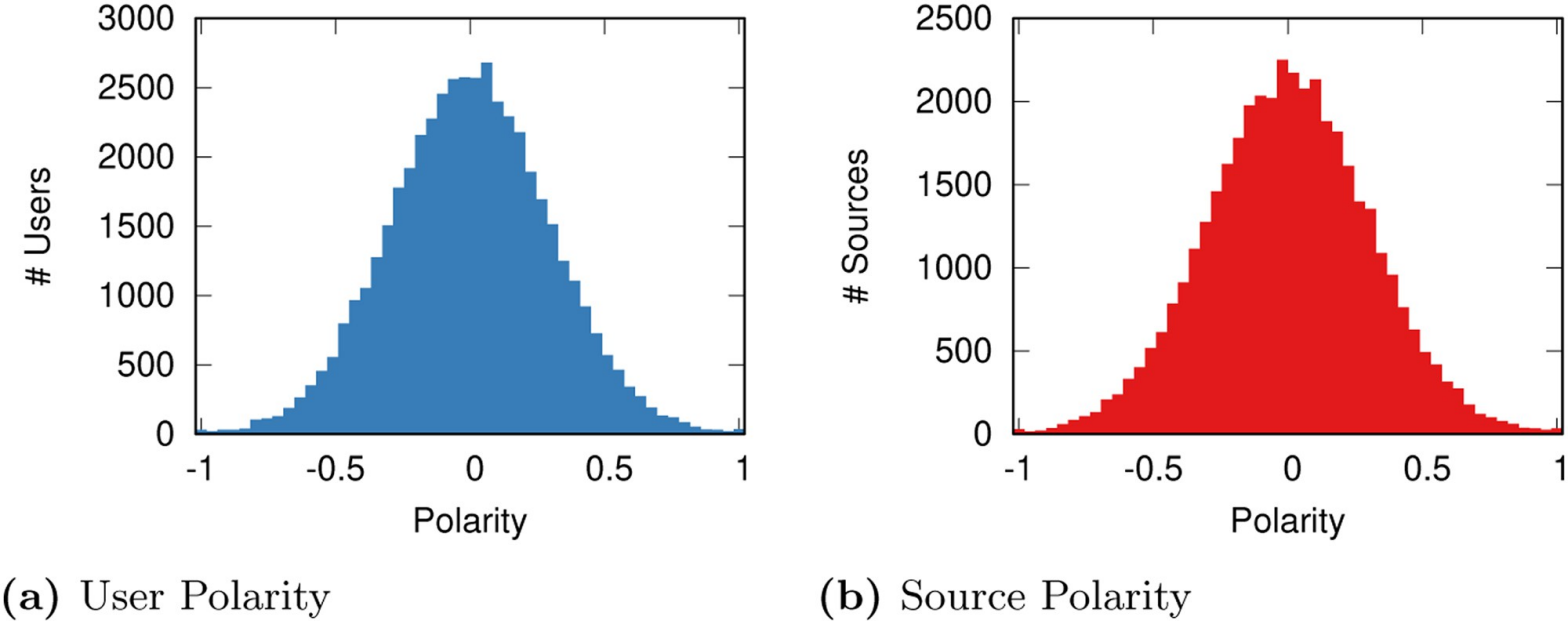
User polarity. Source Polarity. The number of agents (y axis) with a given polarity value (x axis). (a) User Polarity. (b) Source Polarity.

The model has two agents: users and news sources. Both have a polarity value (*p*_*s*_ and *p*_*u*_, respectively) which distributes normally between + 1 and −1—most users/sources are moderates and extremists are progressively more and more rare, see Fig 1. We ensure this by extracting the values from a normal distribution with average equal to zero and standard deviation equal to 1, and then dividing it by its maximum absolute value.

This polarity distribution is supported by studies in the literature [40, 41], although caution should be used if we want to generalize our results to different societies. In such studies, the plurality of people interviewed either felt they were moderate or they did not know what their orientation was—which here we assume being effectively equivalent to being a moderate.

News sources also have a truthfulness value *t*_*s*_ that is between 0 and + 1, showing how trustworthy they are. Truthfulness is correlated with polarity—neutral sources are less biased –, and distributes lognormally—a plurality of news sources are trustworthy. We support these statements with an analysis of real world trustworthiness data in the S1 File.

**Fig 2:**
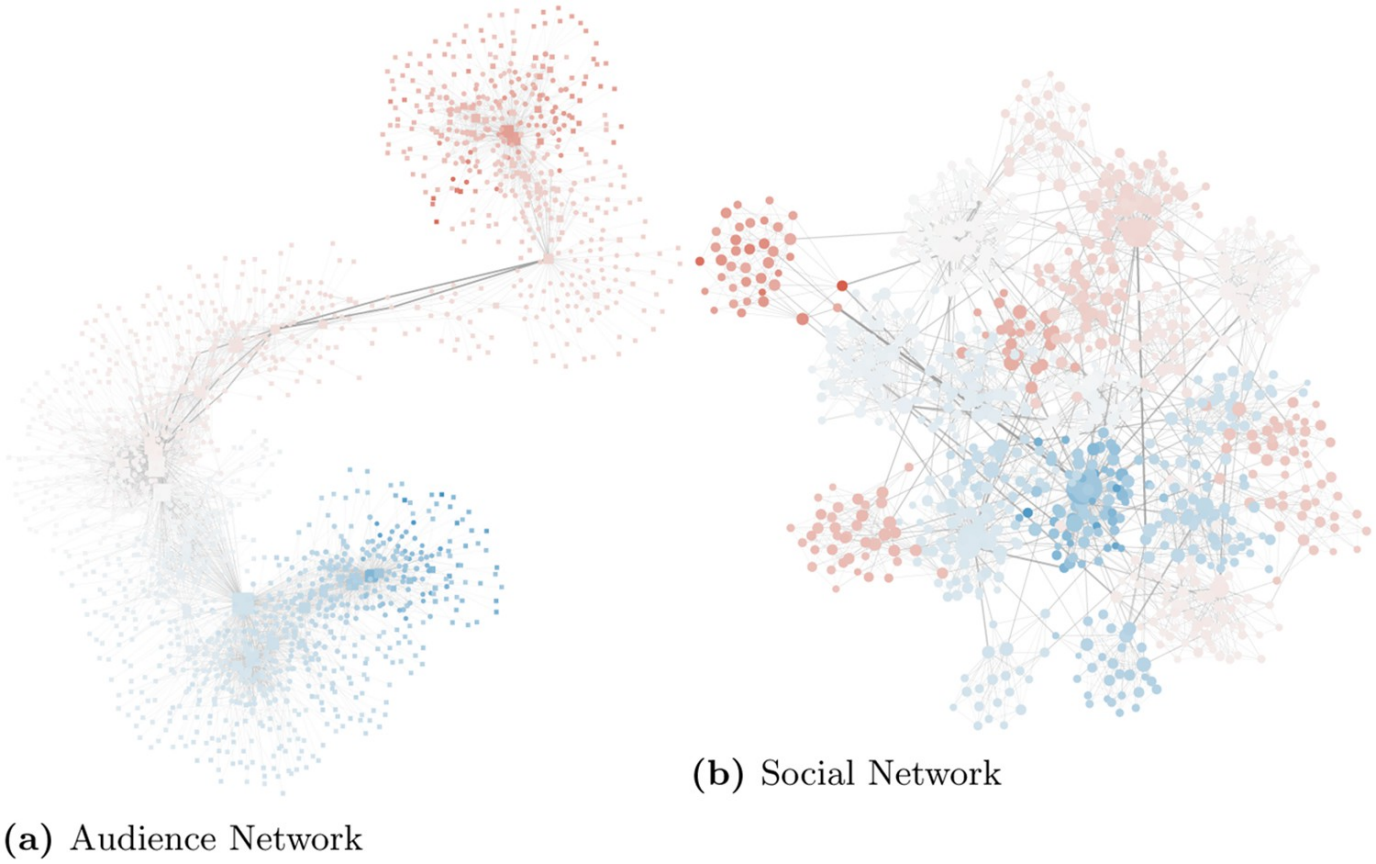
The audience and social network. Each circle is a user and each square is a news source. The colors of circles and squares represent their polarity from −1 (blue) to +1 (red). Each user is connected to the news sources they follow in the audience network and to their friends in the social network. The size of the node is proportional to their degree. Edge thickness and opacity proportional to the edge betweenness. (a) Audience Network. (b) Social Network.

Users connect to news sources in a bipartite audience network (Fig 2(a)). Sources have a broad degree distribution in this network, with few hubs and many sources with low degree. The source degree distribution comes from real world data collected on Facebook. Users tend to connect with sources with the minimum possible polarity difference, modeling selective exposure [42, 43]. Users also connect to each other in a social network (Fig 2(b)), generated with an LFR benchmark [44]—ensuring clustering, small-world, community structure, and power law degree distribution. The social network has a positive polarity homophily: users in the same community tend to have similar polarity values [45].

News sources produce news items with a polarity and truth value equal to their own: *p*_*i*_ = *p*_*s*_ and *t*_*i*_ = *t*_*s*_. Users can see a news item *i* if either they are directly following the source producing it in the audience network, or if they are friends with a user resharing it. If *i* is sufficiently truthful and close to their polarity they will reshare it, while if it is too untruthful and/or far from their polarity they will flag it.

The acceptability *f*_*i*,*u*_ of item *i* for user *u* is directly proportional to the truthfulness *t*_*i*_ of *i*—more truthful items are more acceptable –, and inversely proportional to the polarity distance between *i* and *u*—the more different the worldview of the user with the one of the source the less acceptable its news items are –:

$$\begin{array}{c} f_{i,u}=\frac{t_{i}}{|p_{i}-p_{u}|}. \end{array}$$

For convenience, we transform *f*_*i*,*u*_ into a item-user distance, rather than a similarity, and we normalize it so that it takes values between 0 and 1:

$$\begin{array}{c} \bar{f_{i,u}}=1-\frac{f_{i,u}}{f_{i,u}+1}. \end{array}$$

If $\bar{f_{i,u}}>\phi$, then the news item is too far from the user and they will flag it; if $\bar{f_{i,u}}<\rho$, then the news item is very similar to the user and they will reshare it. *ϕ* and *ρ* are the two parameters of the model and we impose that *ρ* ≤ *ϕ*, otherwise users would flag news items they like enough to reshare. If $\rho \leq \bar{f_{i,u}}\leq \phi$, the user will do nothing.

*ρ* indicates reshareability: the higher *ρ*, the more news items are reshared. *ϕ* indicates tolerance: the higher *ϕ* the fewer news items are flagged—it takes a larger and larger polarity difference and lower and lower truthfulness values to be flagged. *ϕ* is the parameter of main interest in this paper, as *ρ* simply changes the number of articles in circulation and the length of the resharing cascades. For this reason, hereafter we fix *ρ* = 0.08 following [16], unless otherwise specified.

### Main variant: Relative model

In the Bipolar model, the tolerance *ϕ* is equal for everybody. However, an interesting question arises when we allow a group of users to have a lower tolerance. We thus create the Relative model as an evolution of the Bipolar model.

In the Relative model, we divide users in three clusters: left (*p*_*u*_ < −0.2), moderates (−0.2 ≤ *p*_*u*_ ≤ 0.2), and right (*p*_*u*_ > 0.2). The left and right clusters are roughly equally populated, both including around 22% of users. The moderate cluster is the largest of the three, encompassing more than 55% of all users.

We assign a tolerance *ϕ*_*r*_ to the right users. Then, the left users have a tolerance of *ϕ*_*l*_ = *δϕ*_*r*_. The moderates are assumed to be the most tolerant and thus will always assume as their *ϕ* the highest between *ϕ*_*l*_ and *ϕ*_*r*_. *δ* can be higher or lower than 1, thus allowing the left users to be more or less tolerant than the right users.

There are alternative ways to simulate differential tolerance. For instance, we could have a Subtraction model with *ϕ*_*l*_ = *ϕ*_*r*_ − *δ*, or a Fixed model where we fix *ϕ*_*l*_ = 0.1 and vary *ϕ*_*r*_. For specifically chosen values of *δ*, the Subtraction and the Relative models are equivalent. Thus, we include simulations involving the Subtraction model and for different values of *δ* in the S1 File.

### Relative model with moving sources

Sources are not passive agents in the real world: they respond to incentives in a rational way. We encode this in a variant of the Relative model. Once users have flagged the sources, sources apply a simple gradient descent algorithm. They start from their initial polarity level and they follow the slope of the flag probability distribution. This effectively searches for a local minimum in the flag distribution: the polarity value that is can be reached by following the incentives imposed by the users’ flags.

The main output of this model is the new polarity distribution among the sources. Note that we can apply this extension also to the original Bipolar model, which can be useful to compare substantial differences between that model and the Relative one.

### Relative model with moving users

As we support in the introduction, users are also not static agents. When they observe members of the out-group applying a lower tolerance strategy than their own, they might feel the need to retaliate to defend their identity, which is entangled with their belonging to an in-group.

We design an iterative process, which we encode in a Relative model with moving users. This is an extension of the Relative model with moving sources, as also in this variant sources can change their polarity.

Let’s say we start in a very tolerant society with *ϕ*_*l*_ = 0.9. At each step, users on the opposite side will perform a grid search to determine their *ϕ*_*r*_ tolerance. They search in the [*ϕ*_*l*_ − 0.2: *ϕ*_*l*_+ 0.2] range—we assume that it is not possible to perform dramatic tolerance jumps in one movement. Users in the right cluster will settle on the *ϕ*_*r*_ value that results in the sources skewing the most to the right.

Once this happens, the users on the left respond with the same algorithm, this time searching for their new *ϕ*_*l*_ in the [*ϕ*_*r*_ − 0.2: *ϕ*_*r*_+ 0.2] range, using the new *ϕ*_*r*_ as a guidance. At each subsequent step, users in the two clusters take turns in updating their own *ϕ*_*x*_ value to attract news sources to their side. The process stops when neither side can make an advantageous move any more.

Note that a side might end up exploring a *ϕ* value lower than 0.08, which is our default value for *ρ*. Since we must ensure that *ρ* ≤ *ϕ*, when a side explores low *ϕ* values we always make sure to lower *ρ* accordingly. For instance, if the left users want to set *ϕ*_*l*_ = 0.025, then we will also impose that *ρ* = 0.025.

## Results

In this section we report the results of our agent based models. Each figure in this section is the aggregation of 30 independent runs, made to smooth out random fluctuations.

### Flag distribution

**Fig 3:**
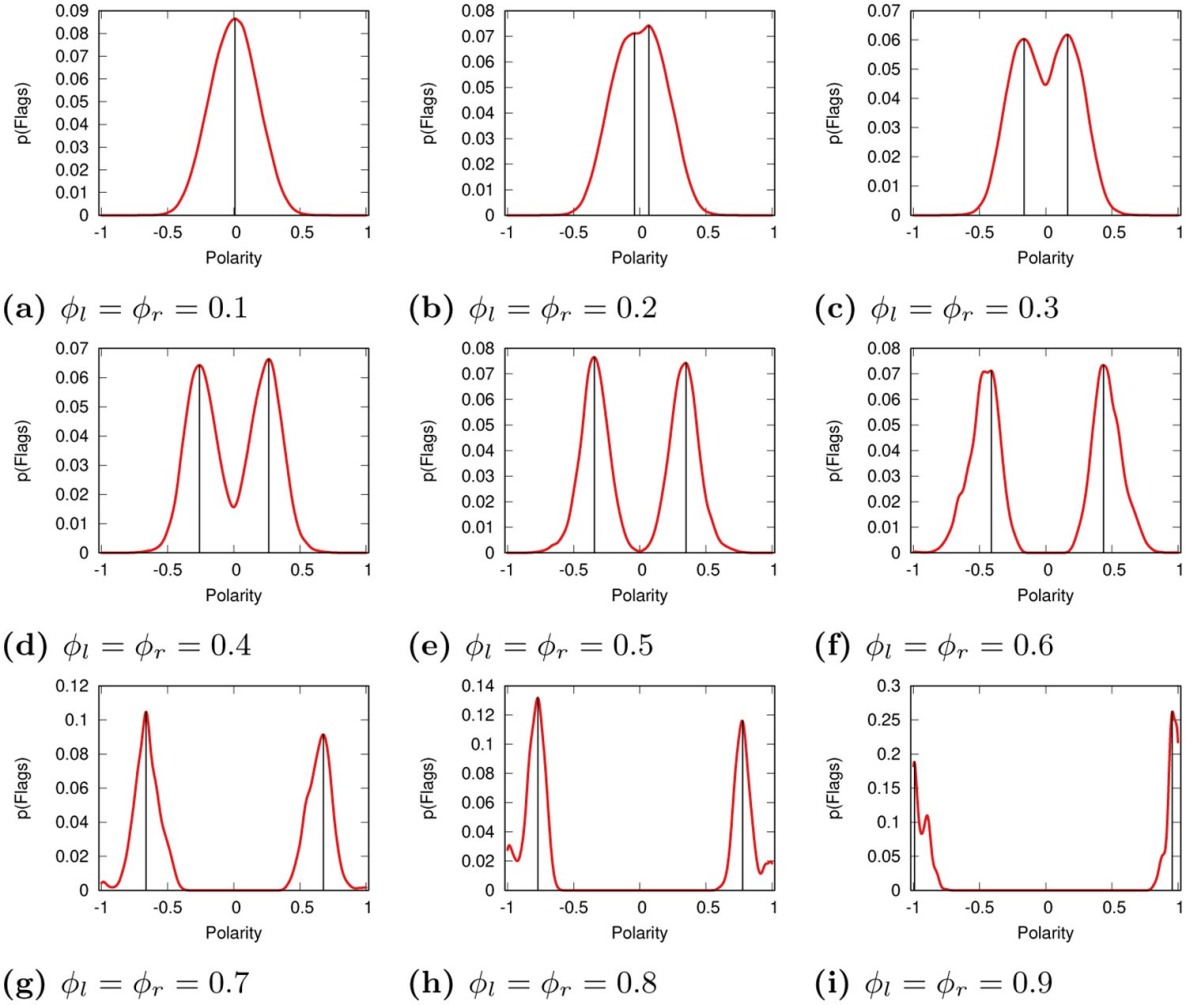
The flag distributions in the Bipolar model for varying levels of tolerance (*ϕ_l_* and *ϕ_r_*). The plot reports the probability that a flag (y axis) will be assigned to a source with a given polarity (x axis). Red lines for KDE estimation, the black thin lines identify the most prominent peaks on the left and the right of zero polarity (if any). (a) *ϕ*_*l*_ = *ϕ*_*r*_ = 0.1. (b) *ϕ*_*l*_ = *ϕ*_*r*_ = 0.2. (c) *ϕ*_*l*_ = *ϕ*_*r*_ = 0.3. (d) *ϕ*_*l*_ = *ϕ*_*r*_ = 0.4. (e) *ϕ*_*l*_ = *ϕ*_*r*_ = 0.5. (f) *ϕ*_*l*_ = *ϕ*_*r*_ = 0.6. (g) *ϕ*_*l*_ = *ϕ*_*r*_ = 0.7. (h) *ϕ*_*l*_ = *ϕ*_*r*_ = 0.8. (i) *ϕ*_*l*_ = *ϕ*_*r*_ = 0.9.

We start by looking at the distributions of the flags generated in the system for different values of *ϕ* in the Bipolar model. Fig 3 reports these distributions and it is a faithful reproduction of one of the figures in [16]. In the Bipolar model there is nothing distinguishing left and right users, and thus the flag distributions are almost perfectly symmetrical, minus the random fluctuations due to different random initial conditions. We confirm the original result that low values of tolerance generate flags mostly for neutral, truthful reporting, and only in presence of high tolerance we see most flags going to the news sources producing polarizing and untruthful items. A high tolerance society thus has better outcomes, since it does not penalize truthful and neutral reporting.

**Fig 4:**
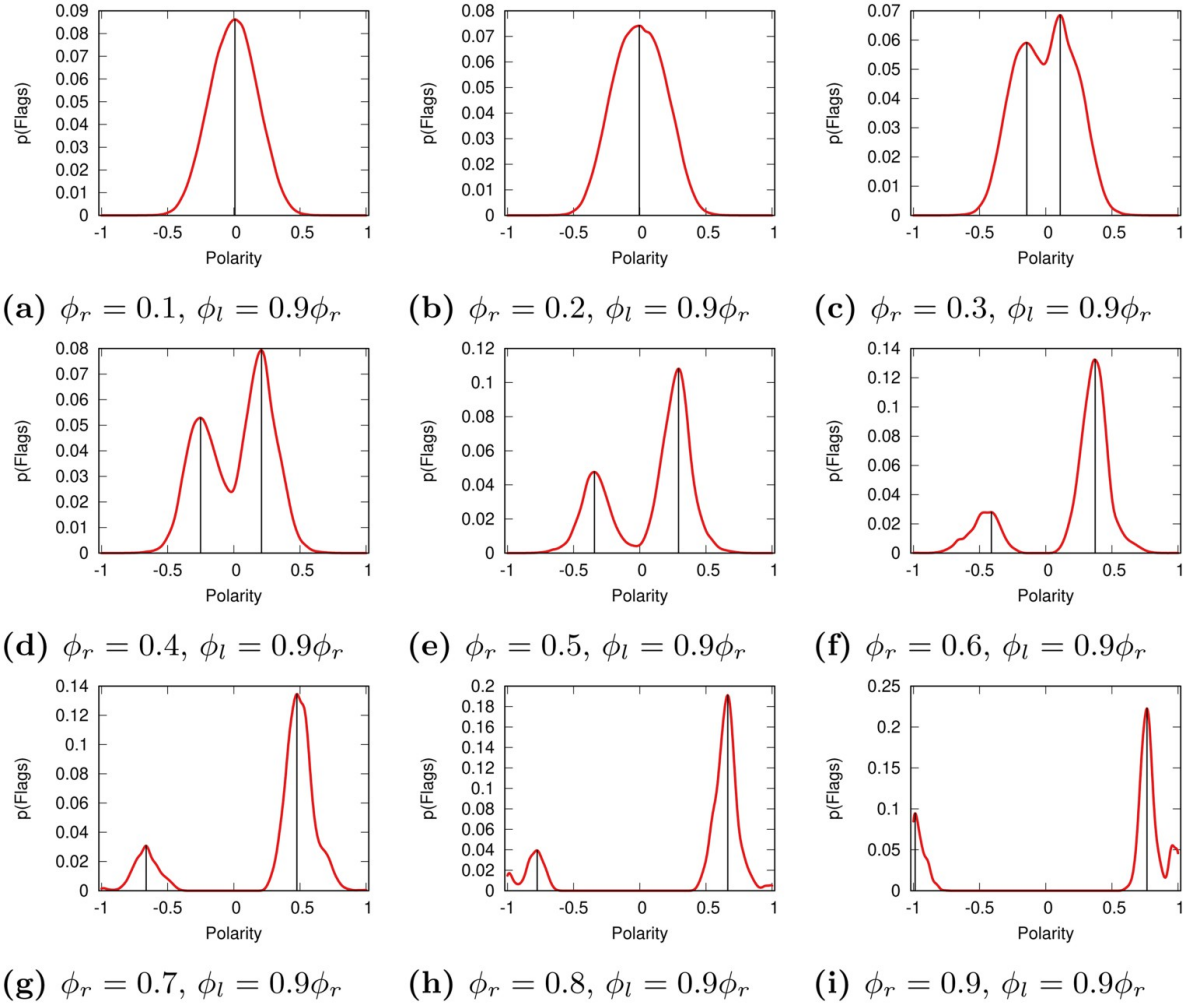
The flag distributions in the Relative model for varying levels of tolerance *ϕ*_*r*_ and fixing *ϕ*_*l*_ = 0.9 *ϕ*_*r*_. Same legend as Fig 3. (a) *ϕ*_*r*_ = 0.1, *ϕ*_*l*_ = 0.9*ϕ*_*r*_. (b) *ϕ*_*r*_ = 0.2, *ϕ*_*l*_ = 0.9*ϕ*_*r*_. (c) *ϕ*_*r*_ = 0.3, *ϕ*_*l*_ = 0.9*ϕ*_*r*_. (d) *ϕ*_*r*_ = 0.4, *ϕ*_*l*_ = 0.9*ϕ*_*r*_. (e) *ϕ*_*r*_ = 0.5, *ϕ*_*l*_ = 0.9*ϕ*_*r*_. (f) *ϕ*_*r*_ = 0.6, *ϕ*_*l*_ = 0.9*ϕ*_*r*_. (g) *ϕ*_*r*_ = 0.7, *ϕ*_*l*_ = 0.9*ϕ*_*r*_. (h) *ϕ*_*r*_ = 0.8, *ϕ*_*l*_ = 0.9*ϕ*_*r*_. (i) *ϕ*_*r*_ = 0.9, *ϕ*_*l*_ = 0.9*ϕ*_*r*_.

For the relative model we set *δ* = 0.9, meaning that the left users are less tolerant than the right users—we test more values for *δ* in the S1 File. Such operation breaks the symmetry in the flag distributions in non-trivial ways—as Fig 4 shows. If tolerance was low to begin with (*ϕ*_*r*_ ≤ 0.2) nothing changes. However, for more tolerant societies (*ϕ*_*r*_ ≥ 0.3), we see that the news sources on the right end of the spectrum attract more flags than news sources on the left. This is expected, since it is the intolerant users in the left cluster that generate these flags. This effect is noticeable even though the users in the left cluster are a small minority—smaller than 25% in fact.

We can quantify this asymmetry by locating the flag peaks for the two distributions, which we report in Table 1. The table only reports the coordinates of the peaks when there are two distinct ones in the Relative model—thus excluding *ϕ*_*r*_ ≤ 0.2. In the Relative model, the right peak is always closer to zero than the left peak, meaning that the flags go to more moderate sources (Table 1(b)). Moreover, the right peak is always higher than the left peak, meaning that more flags are being assigned to more moderate right sources than to less moderate left sources. This is not the case for the Bipolar model (Table 1(a))—excluding minor random fluctuations.

**Table 1:** The coordinates for the peaks for the (a) Bipolar and (b) Relative models.

**(a)** Bipolar				
*ϕ*_*r*_	Left x	Left y	Right x	Right y
0.3	-0.164	0.060	0.164	0.062
0.4	-0.260	0.064	0.264	0.066
0.5	-0.344	0.076	0.348	0.074
0.6	-0.412	0.071	0.436	0.073
0.7	-0.664	0.104	0.676	0.091
0.8	-0.772	0.132	0.776	0.116
0.9	-0.992	0.188	0.956	0.262
**(b)** Relative				
*ϕ*_*r*_	Left x	Left y	Right x	Right y
0.3	-0.144	0.059	0.108	0.068
0.4	-0.252	0.053	0.208	0.079
0.5	-0.344	0.048	0.292	0.108
0.6	-0.412	0.028	0.372	0.132
0.7	-0.664	0.031	0.476	0.134
0.8	-0.776	0.039	0.664	0.191
0.9	-0.988	0.095	0.764	0.222

Note that this pattern is supported by most *δ* values and in the Subtraction model as well. Moreover, a Relative model initialized with different—less realistic—conditions, like random networks with no homophily, fails to achieve these clear results. The S1 File contains the analysis supporting both statements.

### Pressure to conform

**Fig 5:**
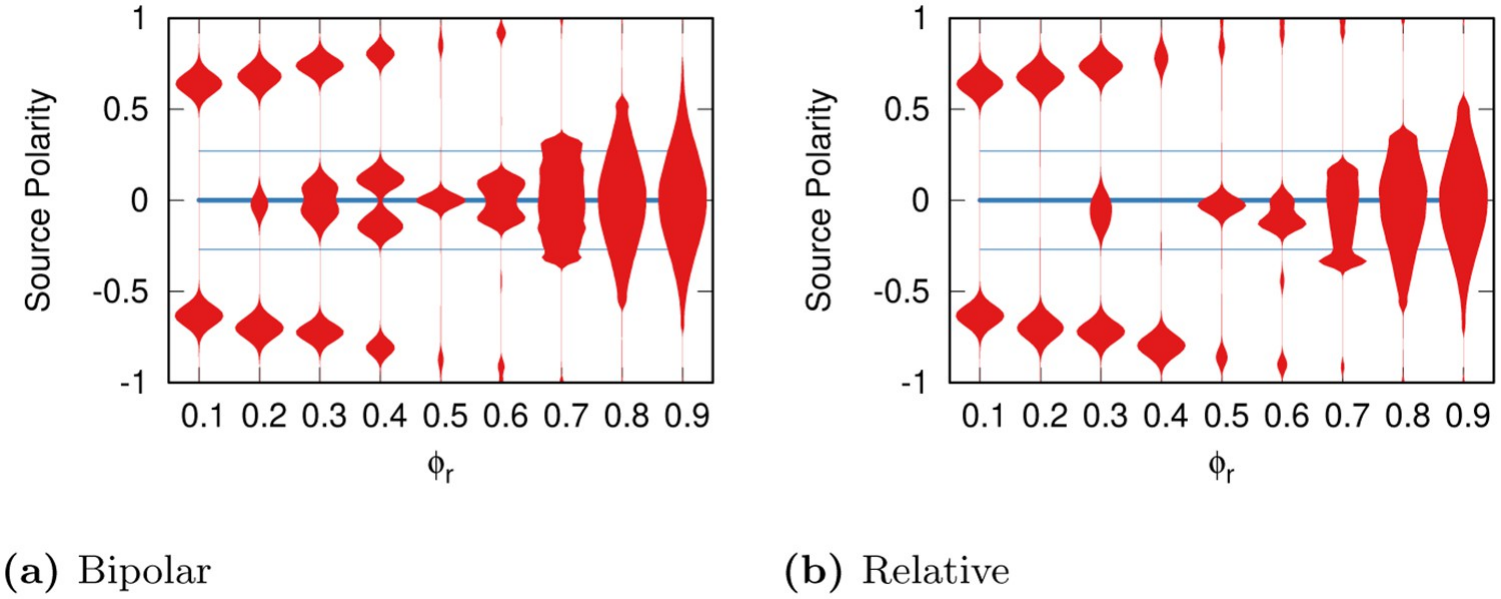
The polarity distributions (y axis) for sources after gradient descent in the (a) Bipolar and (b) Relative models, per value of *ϕ_r_* (x axis). The thick and thin blue lines represent the initial average polarity mean and standard deviation of the sources before gradient descent, respectively.

It is reasonable to assume that a news source wants to minimize the number of flags it receives. A high number of flags would cause the social media platform to hurt the reach of the news source in many ways, even coming to a total ban. Following this logic, we test what happens in the Relative model with moving sources, in which sources seek for a flagging local minimum that is the closest to their own initial polarity. Fig 5 shows the resulting polarity distribution for sources for each value of *ϕ*_*r*_—again fixing *δ* = 0.9.

Since there is nothing to distinguish the left and right side of the polarity spectrum in the Bipolar model, the sources will distribute symmetrically around zero (Fig 5(a)), just like the corresponding distributions of flags in Fig 3. For low tolerance scenarios this implies the sources fleeing from neutrality and clustering on medium/extreme positions on either side; while for high tolerance scenarios the sources will agglomerate in the neutral portion of the polarity spectrum. A high tolerance society thus has better outcomes because it does not encourage sources to increase their polarization.

In the Relative model with moving sources the symmetry is broken around *ϕ*_*r*_ = 0.3 and beyond. In these cases, sources tend to skew into the left portion of the polarity spectrum. This is why we say that intolerance is attractive: by being less tolerant, left-leaning users can cause sources to adopt their polarity values, to avoid being flagged.

### Intolerance Arms’ Race

Since users on one side can attract news sources by being intolerant, users on the other side might want to copy the strategy to attract the news sources to their side in turn. We encode this in the Relative model with moving users.

**Fig 6:**
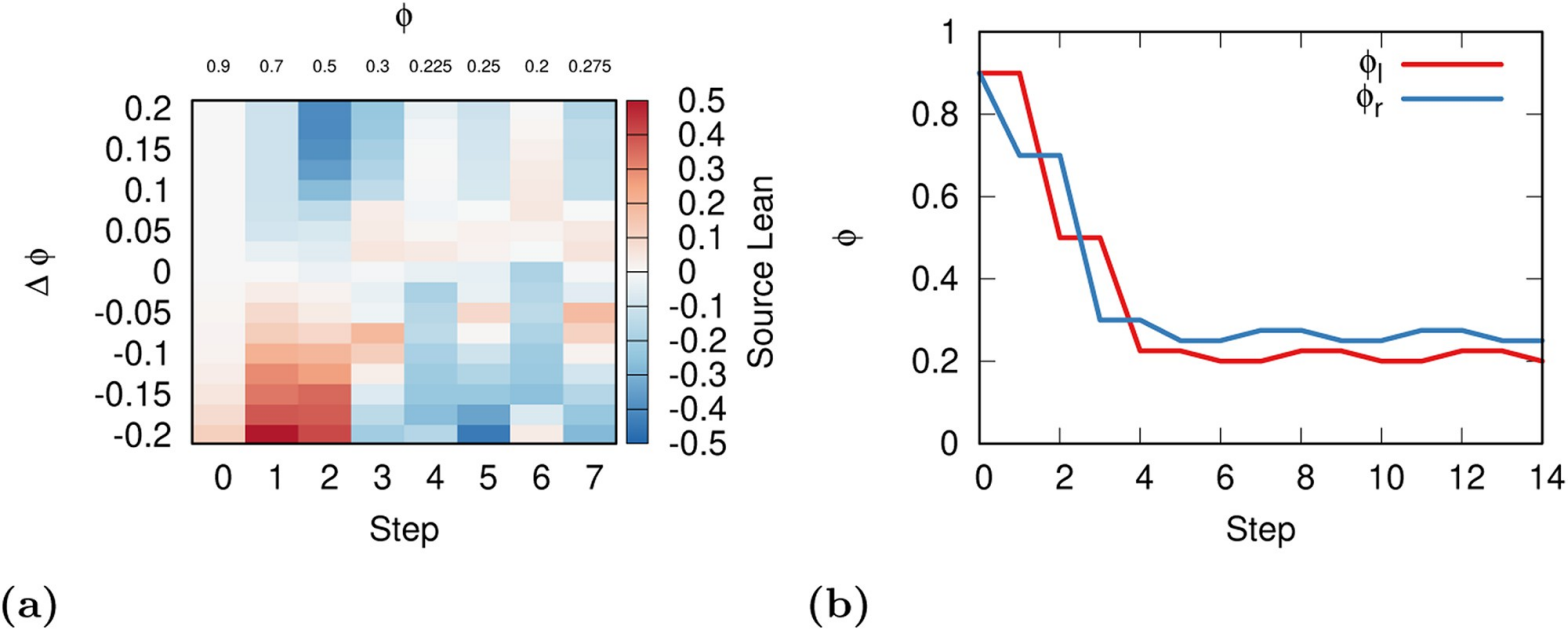
a. (a) For each step of the simulation (x axis) the amount of source polarity attraction (color) for a given change in tolerance with respect to the tolerance of the other side (y axis). Red indicates an attraction of the sources, blue a repulsion. Odd steps for changes in *ϕ*_*l*_, even steps for changes in *ϕ*_*r*_. Above each column we report the value of the opposite (fixed) *ϕ* for each step. (b) The evolution of *ϕ*_*l*_ (red) and *ϕ*_*r*_ (blue) for each step of our simulation (x axis).

Fig 6 shows what are the consequences of living in a world following such a model. From the figure we see that, in the initial steps, it pays off to both left and right users to jump to the lowest possible tolerance value that they are allowed to reach. *ϕ* quickly drops from 0.9 to 0.3 in few steps. Then, *ϕ* plateaus to a cyclic recurring state oscillating between 0.2 and 0.225 for *ϕ*_*l*_, and between 0.25 and 0.275 for *ϕ*_*r*_. This slight asymmetry for left and right users is due to small random fluctuations in our initialization that makes left users slightly more populous (22.5% vs 21.9%).

In the Relative model with moving users, high tolerance scenarios are out of equilibrium: the system will tend to converge to low tolerance regimes. In our model, the value of *ϕ* supporting a stable system is between 0.2 and 0.275. This is contained in the *ϕ* value interval that, in previous work, was able to better approximate real world flagging behavior in Facebook. For *ρ* = 0.08, which is what we use in this paper, *ϕ* needs to be between 0.1 and 0.3 to correctly approximate the relationship between a source’s popularity and the number of flags it receives [16]. This result suggests that Facebook could have reached a low tolerance equilibrium with a process similar to the one we outline in this section.

## Discussion

The results of our simulations suggest a number of interesting insights about flagging misinformation on social media in the presence of biased users with different levels of tolerance for opposing points of view:

- News sources at the opposite side of the intolerant users will be flagged more;
- If sources want to minimize the number of received flags, they will be attracted to the intolerant side;
- If users want to attract news sources to their side, high-tolerance systems are out of equilibrium and users will naturally converge to low tolerance values;
- The equilibrium state of the model is in the tolerance parameter values that better reproduce real world data, as identified in previous works.

It is important to notice that these are only suggestive results and they call for more research to be done, if we want to confirm them. Specifically, everything is based on simulations from an agent-based model. Thus, our results are only as good as the assumptions baked in the model. We can identify several pathways that call for more future works.

First, the baseline Bipolar model might not be a perfectly accurate description of reality. However, among the models approximating online flagging behavior, it is the only one including polarity homophily in both user-user and user-source networks, it has a realistic social network topology, source popularity distribution, and uses evidence-supported polarity distributions. Thus, we think it is the most reasonable approximation we have.

Second, the Relative model hinges on the assumption that users on different sides of the polarity spectrum might have different tolerance levels. While we find some literature support for this assumption [19–21], it is worth noting that none of these papers is about flagging content on social media. Thus, we would need an experiment closer to our assumption to confirm it.

Third, we assume that news sources might want to minimize the number of flags received on social media. While this seems to be a common sense assumption, it is still to be tested. We do see a rise in partisan news sources [46], but the arrow of causation is not clear: are these news sources emerging because their authors are trying to game the flagging system on social media in presence of partisan users, or are they the ones creating partisanship in the user base? In the former case we would have user flagging shaping source polarity which would support our assumption. In the latter case we would have news sources causing user polarization, which would make our assumption harder to justify. Determining which is which is an extremely difficult problem, but the conclusion seems to be that there is no firm evidence that news sources are making users more polarized, at least in the US [47].

Fourth, our model does not include fact-checkers. It is entirely possible that, once news items get disproportionally flagged for partisan reasons, the fact-checkers can correct the mistake and refuse to remove the factual article. In this sense, the presence of fact checkers will help the system to be more fair. The potential issues arising from our models would thus need to be interpreted as overestimates. However, fact-checkers can only help to a point, for two reasons. First, fact-checkers can only counteract false positives: news items that were flagged but should not have. No fact-checker can prevent the spread of misinformation in a community that embraced the misinformation and is not flagging it. Such misinformation can still increase polarization. Second, fact-checkers are as human as users and can be biased themselves [48]. Moreover, fact-checkers are only effective if they have the trust of the users. This might not be the case, as researchers found that users are more likely to believe fact-checkers if they confirm the opinion the user already had before the fact-checking [49].

Fifth, our last experiment rests on the assumption that users can change their tolerance—whether deliberately or subconsciously—for strategic reasons: to shift the news sources to their side. Such action would be reasonable for a rational partisan agent, but we still need empirical support for it.

Finally, the model is not necessarily exclusively talking about political polarization. If one can define meaningful *p*_*s*_ and *p*_*u*_ values for other information-producing processes—e.g. sport results –, the model could inform us about other non-political phenomena—in our example, sport rivalries. In this scenario, inferring whether *ϕ*_*l*_ = *ϕ*_*r*_—and/or whether their value is high or low—from real world data would be the key to investigate whether intolerance spirals happen in different scenarios.

## Supporting information

S1 File Additional analysis supporting the findings in the main paper, including supporting figures, tables, and references. (PDF)
