# Supplementary material for: A potential mechanism for low tolerance feedback loops in social media flagging systems
Source: PLoS One. 2022 May 26;17(5):e0268270. doi: 10.1371/journal.pone.0268270 (PMC9135209; doi:10.1371/journal.pone.0268270)
Supplement: S1 File — (PDF) [file pone.0268270.s001.pdf]

Supporting Information for “A Potential Mechanism for Low Tolerance Feedback Loops in Social Media Flagging Systems”

Camilla Jung Westermann[1], Michele Coscia[1*]

1 IT University of Copenhagen, Copenhagen, Denmark

* mcos@itu.dk

1 Sensitivity Analysis

In this section we test how robust our results are. We start by analyzing the effect of the models' parameters and then we move onto the effect of the models' starting conditions.

1.1 Parameter Sensitivity

In the main paper, we base our results on the Relative model with $\delta = 0.9$. First, we verify what happens when we vary δ. We remind that δ regulates how distant ϕ_l is from ϕ_r, i.e. $\phi_l = \delta\phi_r$.

Figure S1 shows the distributions of flags for different ϕ_r and δ values. It is a reproduction of Figure 4 in the main paper. We only show the Kernel Density Estimations for clarity. We can see that we confirm the main result of the paper: in the Relative model for $\phi_r \geq 0.3$ there are asymmetric flag peak probabilities, with the right side of polarity attracting more flags.

As δ shrinks, the difference between ϕ_r and ϕ_l grows. The effect is that the left-leaning news sources gets flagged less and less, while the flagging peak for right-leaning sources moves toward zero. We interpret this result later in this section, as it requires more information to be properly understood.

We now turn to considering an alternative model: the Subtraction model. In the Subtraction model, $\phi_l = \phi_r - \delta$. Just like before, we test different values of δ.

Figure S2 shows the distributions of flags for different ϕ_r and δ values – again only showing the Kernel Density Estimations for clarity. Its interpretation is the same as Figure S1. Also in this case, we can confirm the main result of the paper: in the Subtraction model for $\phi_r \geq 0.4$ there are asymmetric flag peak probabilities, with the right side of polarity attracting more flags.

As δ grows, the difference between ϕ_r and ϕ_l grows more slowly than with the Relative model. This is because in the Relative model we test larger differences (varying δ between 0.1 and 0.9) than in the Subtraction model (varying δ between 0.025 and 0.225). Please note that the values of δ are not directly comparable across models, because they depend on ϕ_r's value. For instance, if $\phi_r = 0.1$, then a $\delta = 0.5$ in the Relative model corresponds to a $\delta = 0.05$ in the Subtraction model (because they both result in $\phi_l = 0.05$). Vice versa, if $\phi_r = 0.9$, then a $\delta = 0.5$ in the Relative model corresponds to a $\delta = 0.45$ in the Subtraction model (because they both result in $\phi_l = 0.45$). This is the reason why we test a wider interval for δ in the Relative model than in the Subtraction model.

Also in this case, we see that for larger and larger δ differences, the right peak tends to move towards zero. Note that in the Relative model high δ means little difference, while the opposite is true for the Subtraction model. This is the reason why the color

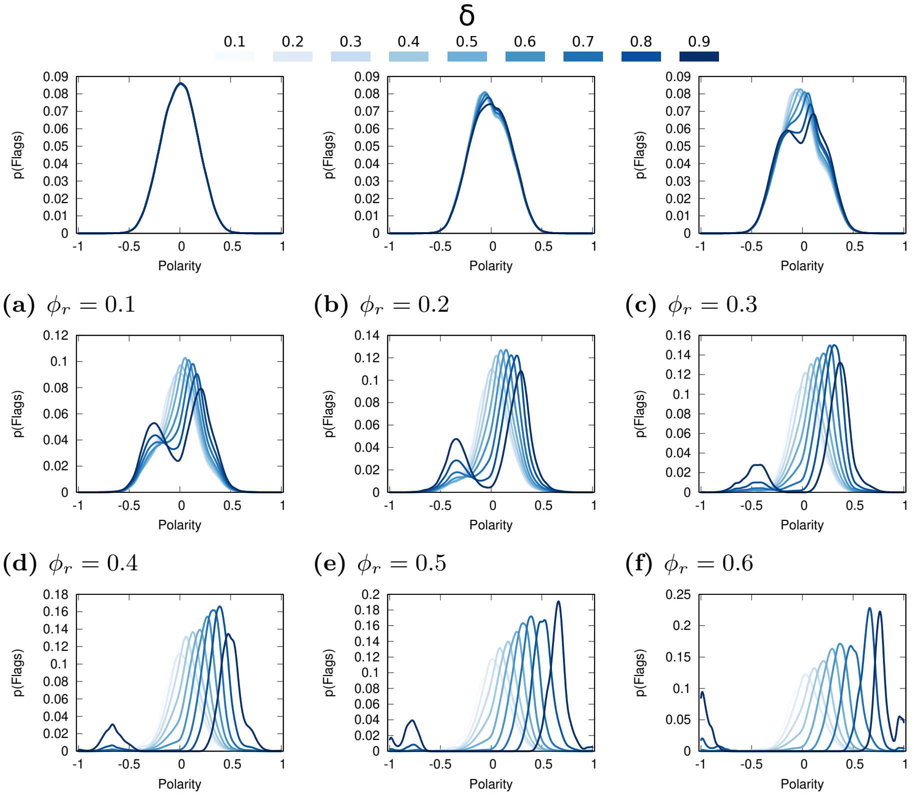

(a) $\phi_r = 0.1$ **(b)** $\phi_r = 0.2$ **(c)** $\phi_r = 0.3$

(d) $\phi_r = 0.4$ **(e)** $\phi_r = 0.5$ **(f)** $\phi_r = 0.6$

(g) $\phi_r = 0.7$ **(h)** $\phi_r = 0.8$ **(i)** $\phi_r = 0.9$

Fig S1. The flag distributions in the Relative model for varying levels of tolerance ϕ_r and fixing $\phi_l = \delta\phi_r$, with δ varying from 0.1 (light blue) to 0.9 (dark blue). The plot reports the probability that a flag (y axis) will be assigned to a source with a given polarity (x axis).

gradients in Figures S1 and S2 go in opposite directions. We now perform an additional analysis to properly interpret this observation.

Figure S3 shows the average source polarity once we perform the gradient descent by using the flag distributions we see in Figures S1 and S2. Figure S3(a) refers to the Relative model, while Figure S3(b) refers to the Subtraction model. These figures are an aggregation of Figure 5 in the main paper: rather than showing the full distributions as we do in the main paper, here we only show the mean of the distribution.

We can see that, in both models, for most values of δ the left users are able to shift toward the left (negative) the average polarity of the sources. The only exception is when we have a large difference (i.e. ϕ_l is much smaller than ϕ_r) in scenario where ϕ_r is already low (≤ 0.3) to begin with. This confirms one of the takeaway of the paper: there is a non-zero bottom for intolerance. When the system reaches a low tolerance value, being less tolerant than this threshold is counterproductive.

Also note how there is a sweet spot for δ in the Relative model that follows ϕ_r. For instance, the best value for $\phi_r = 0.8$ is $\delta = 0.6$. Values either higher of lower than 0.6 for δ will result in a weaker attraction of sources. This confirms that the trivial interpretation of our results ("the lowest tolerance the best") is incorrect. It also shows how the optimal flag distribution in Figures S1 and S2 does not have a right peak in the middle, as one might naively expect, but still needs to be decisively on the right. The reason is that a peak in the middle will push a significant portion of neutral sources to

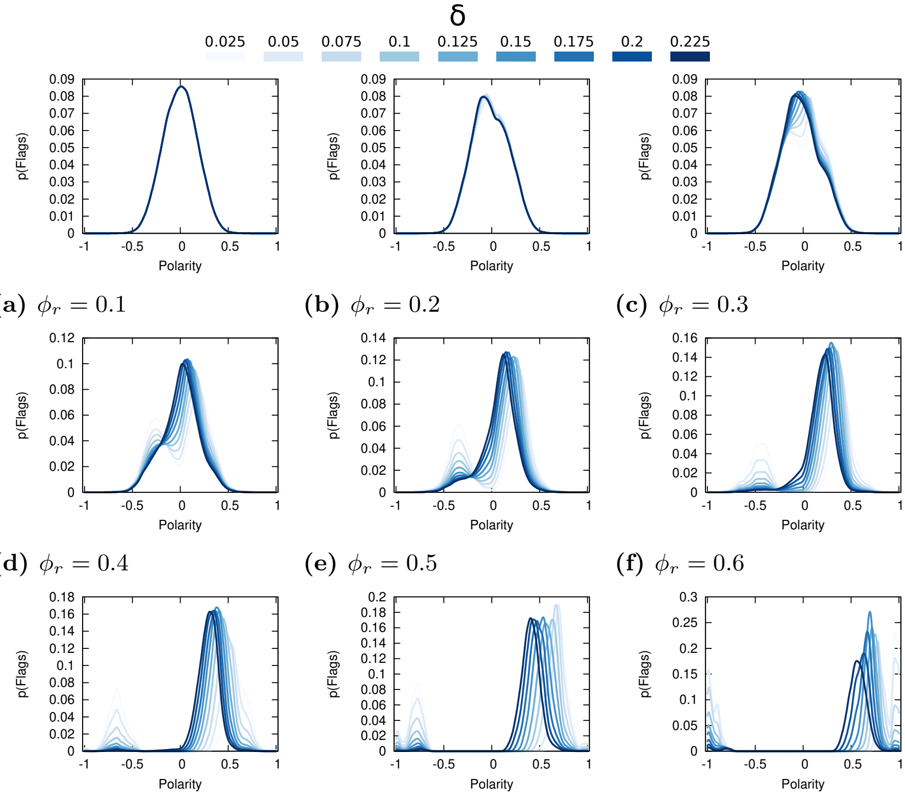

(a) $\phi_r = 0.1$ **(b)** $\phi_r = 0.2$ **(c)** $\phi_r = 0.3$

(d) $\phi_r = 0.4$ **(e)** $\phi_r = 0.5$ **(f)** $\phi_r = 0.6$

(g) $\phi_r = 0.7$ **(h)** $\phi_r = 0.8$ **(i)** $\phi_r = 0.9$

Fig S2. The flag distributions in the Subtraction model for varying levels of tolerance ϕ_r and fixing $\phi_l = \phi_r - \delta$, with δ varying from 0.025 (light blue) to 0.225 (dark blue). The plot reports the probability that a flag (y axis) will be assigned to a source with a given polarity (x axis).

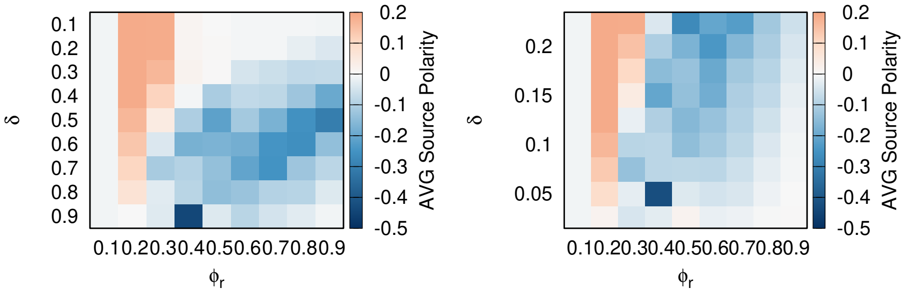

(a) Relative **(b)** Subtraction

Fig S3. The average source polarity after gradient descent in the (a) Relative and (b) Subtraction models per value of ϕ_r (x axis) and δ (y axis). y-axis flipped in (a) to make the comparisons between the figures easier.

the right, rather than to the left.

Note how the same could be said for the Subtraction model, if we had chosen a wider range of δ values to test. The pattern we can see looks remarkably similar. In fact, as noted in the main paper and shown above, it is always possible to find a pair of Relative and Subtraction δ values that would result in the same ϕ_r-ϕ_l pairing.

1.2 Initial Conditions

In the main paper, we initialize the models by using a realistic distribution of polarity following homophily, and a realistic shape of the social and audience networks. In this section we test what happens when polarity and social/audience connections are distributed randomly. Just like in the main paper, we perform 30 independent initializations and we report the aggregated results.

In this Relative Random model, each user assumes a value from the polarity distribution that is independent from the ones of its neighbors. The social network is an Erdos-Renyi random graph with the same number of nodes and roughly the same number of edges as the original network. The audience network is the same, with the additional constraint of being a bipartite user-news source network. We ensure that the networks are connected in a single connected component.

ϕ_r	Left x	Left y	Right x	Right y
0.3	-0.144	0.059	0.108	0.068
0.4	-0.252	0.053	0.208	0.079
0.5	-0.344	0.048	0.292	0.108
0.6	-0.412	0.028	0.372	0.132
0.7	-0.664	0.031	0.476	0.134
0.8	-0.776	0.039	0.664	0.191
0.9	-0.988	0.095	0.764	0.222

(a) Relative

ϕ_r	Left x	Left y	Right x	Right y
0.3	-0.076	0.092	0.084	0.105
0.4	-0.144	0.071	0.152	0.120
0.5	-0.192	0.043	0.188	0.174
0.6	N/A	N/A	0.220	0.231
0.7	-0.72	0.056	0.552	0.077
0.8	-0.832	0.084	0.672	0.087
0.9	-0.932	0.141	0.992	0.154

(b) Relative Random

Table S1. The coordinates for the peaks for the (a) Relative and (b) Relative Random models.

Table S1(a) reports the results from the Relative model in the main paper – it is a reproduction of Table 1(b) in the main paper. The original pattern in the Relative model is that the left peaks are consistently both farther from the 0 point and smaller than the right peaks for any value of ϕ_r.

On the other hand, Table S1(b) shows that this is not the case for the Relative Random model. For low values of ϕ_r, the left and right peaks are roughly equidistant from 0 – the x values are comparable. For high values of ϕ_r, the peak sizes are comparable in their y axis value. Thus we can conclude that the specific realistic initial conditions of the Relative model play a role in augmenting the effect of differential tolerance. If we had random social networks with no homophily, we would not see such an evident and consistent difference in flagging from the opposite sides.

2 News Source Trustworthiness

In the main paper we argue that there is a correlation between the bias of a news source and its trustworthiness – i.e. neutral sources are more trustworthy. We also argue that most news sources are trustworthy. Here we support these claims by analyzing data from `https://mediabiasfactcheck.com/` a website aggregating fact-checking information that has been used in multiple literature studies [1–6].

The website contains information about thousands of news media websites and uses two classifications: their political bias (left, neutral, right) and their level of factual reporting (high, mixed, questionable). We count all the sources that have both pieces of information reported. First, the plurality of sources (46%) have high factual reporting. This is in line with our initialization of the model, where 43% of sources have a t_s score higher than 0.85, which indicates high factual reporting.

We support our claim of correlation between bias and trustworthiness by showing, in Figure S4, that the likelihood of being trustworthy is much higher for neutral sources than for sources with any bias. This is still true if we ignore the left-right distinction:

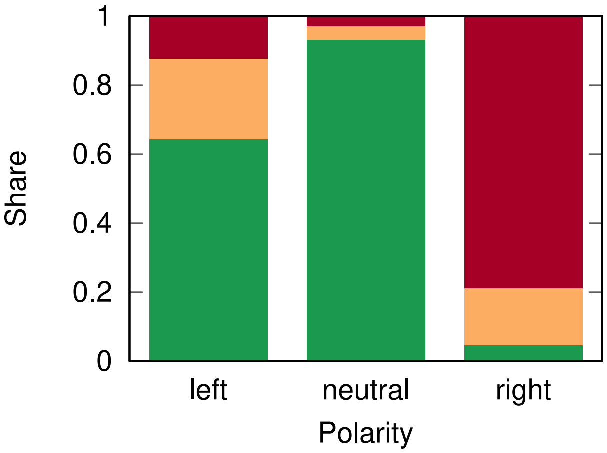

Fig S4. The share of news sources (y axis) with a given factual score (red = questionable, yellow = mixed, green = high) per leaning (x axis).

93% of neutral sources have high factual rating, against only 26% of the sources leaning either left or right.

References

1. Bovet A, Makse HA. Influence of fake news in Twitter during the 2016 US presidential election. Nature communications. 2019;10(1):1–14.

2. Cinelli M, Morales GDF, Galeazzi A, Quattrociocchi W, Starnini M. The echo chamber effect on social media. Proceedings of the National Academy of Sciences. 2021;118(9).

3. Le H, Maragh R, Ekdale B, High A, Havens T, Shafiq Z. Measuring political personalization of Google news search. In: The World Wide Web Conference; 2019. p. 2957–2963.

4. Stefanov P, Darwish K, Atanasov A, Nakov P. Predicting the topical stance and political leaning of media using tweets. In: Proceedings of the 58th Annual Meeting of the Association for Computational Linguistics; 2020. p. 527–537.

5. Naeem SB, Bhatti R. The Covid-19 'infodemic': a new front for information professionals. Health Information & Libraries Journal. 2020;37(3):233–239.

6. Primario S, Borrelli D, Iandoli L, Zollo G, Lipizzi C. Measuring polarization in Twitter enabled in online political conversation: The case of 2016 US presidential election. In: 2017 IEEE International Conference on Information Reuse and Integration (IRI). IEEE; 2017. p. 607–613.
